# A cross-sectional survey: Exploring future healthcare workers' intention to use cannabis through extended theory of planned behavior

**DOI:** 10.3389/fpubh.2022.929016

**Published:** 2022-08-22

**Authors:** Sze Wing Cherelle Ho, Yuen Lung WONG, Pui Hong Chung

**Affiliations:** ^1^The Jockey Club School of Public Health and Primary Care, The Chinese University of Hong Kong, Shatin, Hong Kong SAR, China; ^2^Faculty of Medicine, The Chinese University of Hong Kong, Shatin, Hong Kong SAR, China; ^3^School of Public Health, The University of Hong Kong, Pokfulam, Hong Kong SAR, China

**Keywords:** cannabis, theory of planned behavior, university students, future allied health workers, prevalence, questionnaire, young adult, adolescent

## Abstract

Cannabis is the most extensively abused drug, leading to multiple health burdens such as traffic accidents and psychosis. There is a global wave of legalization of recreational and medical cannabis. This study aimed to understand future healthcare workers' intention to use cannabis through extended Theory of Planned Behavior (TPB). An online cross-sectional survey on cannabis, including validated survey tools and questions on demographics, knowledge, and constructs of the TPB was designed, and distributed during virtual classes in late 2020. Responses were obtained from the Faculty of Medicine of a local university. Nine hundred ninety-six responses were collected, of which 629 were complete and analysed. Age was the only demographic variable associated with cannabis use intention (*p* = 0.029). Respondents with intention had better knowledge of cannabis. All TPB and additional constructs, including perceived behavioral control (COR = 3.44, 95% CI 2.72–4.35, *p* < 0.001), descriptive norm (COR = 2.24, 95% CI 1.81–2.77, *p* < 0.001), injunctive norm (COR = 0.51, 95% CI 0.42–0.61, *p* < 0.001), attitude (COR = 1.23, 95% CI 1.18–1.28, *p* < 0.001), knowledge (COR = 1.08, 95% CI 1.03–1.14), and perceived availability (COR = 2.75, 95% CI 2.22–3.40, *p* < 0.001) were individually associated with intention. In the final multiple logistic regression model adjusted for age, only attitude (AOR = 1.19, 95% CI 1.13–1.25, *p* < 0.001) and perceived availability (*p* = 0.004) showed statistically significant associations with intention. Descriptive norm (standardized coefficient = 0.570) had better explanatory power than the injunctive norm (standardized coefficient = −0.143) in the model. Perceived behavioral control was associated with intention among respondents with negative to neutral attitudes towards cannabis (AOR = 2.48, 95% CI 1.63–3.77, *p* < 0.001), but not among those with positive attitudes. All TPB constructs positively correlated with the intention to use cannabis. Changing the attitudes and perceived control on cannabis use may be useful in preventing cannabis use.

## Introduction

Cannabis use refers to the consumption of cannabis plant products, typically for recreational or medical purposes. The main psychoactive substance in cannabis products is delta-9 tetrahydrocannabinol (THC), which gives users a sense of euphoria and “high.” Another active ingredient is cannabidiol (CBD), which is not psychoactive. Cannabis is the most extensively used illicit drug, and its market size is rapidly expanding (1). There were an estimated 200.4 million users in 2019, approximately accounting for 4.0% of the global population (2). Cannabis also accounts for half of all drug seizures (3). In the United States, 3.5 million people initiated cannabis use in 2019, among which 38.9 per cent are between the ages of 12 and 17 years (4). Sociodemographic analysis shows that young adults aged between 18 and 25, males over age 18, and college graduates have a greater past-year prevalence of cannabis use (4).

Cannabis use is associated with numerous adverse outcomes, notably a heightened risk of psychosis, mood disorders, and cognitive impairments (5–8). Associated systemic effects include respiratory symptoms, and cardiovascular events (9–12). 22.1 million people were diagnosed with cannabis use disorder (CUD) in 2016. The median age of onset for CUD was found to be 22 in a meta-analysis covering Australia, Europe, and the US (13). Other effects include injuries and deaths from motor vehicle accidents (7). Despite the health burden and young ages involved, a wave of cannabis legalization and decriminalisation is underway. It was accompanied by an 18 per cent increase in cannabis use over the past 10 years (14). As of mid-2021, six countries legally permit the possession and use of recreational cannabis. More than 30 nations have decriminalised it, and more than 60 have allowed medical cannabis. Sativex, a cannabis extract with THC and CBD, has been approved for medical use in 27 countries. Epidiolex, which only contains CBD, was also approved for intractable childhood epilepsy. The general perception of cannabis has become less negative. Perceived harmfulness was lowered among adolescents in the US and Europe (15).

In Hong Kong, all cannabis and its psychoactive derivatives, including THC, are illegal (16). Cannabis trafficking and possession/consumption in Hong Kong are criminal offences punishable by a fine of up to USD 636,949 and life imprisonment, and a fine of up to USD 127,388 and imprisonment for seven years, respectively (16). However, pure CBD is not restricted, and its oil products are available on the market (17). The culture seems to disfavour cannabis use, but cannabis is becoming more popular among young adults (18). In general, cannabis use has become more prevalent in Hong Kong, especially among adolescents aged below 21, which account for 49% of local illicit drug users. Those aged 21 or above only account for 9% of users (19, 20). Overall, the growing popularity of cannabis in Hong Kong resembles the global landscape.

Recent studies show that the theory of planned behavior (TPB) could be used to explain cannabis use in different populations (21–24). TPB provides a framework for explaining and predicting human decisions, such as the initiation and termination of addictive behaviors (25, 26). TPB suggested that behavioral intention is the most influential predictor of behavior. Behavioral intention is predicted using attitude (positive or negative evaluations of cannabis use), injunctive norm (perceptions about what significant others think about cannabis use) and perceived behavioral control (perceptions of the ease or difficulty of controlling cannabis use). Meta-analysis have established the model's efficacy in different health behaviors (27, 28). However, it is increasingly shown that the descriptive norm (perception of the extent to which others are engaging in the behavior) is particularly relevant to young adults transitioning to college, suggesting the possibility that the descriptive norm gives TPB a higher predictive power than the injunctive norm (23, 29). Our study assessed whether the TPB model, with the two types of norms included, could explain the intention to use cannabis in Hong Kong.

Ajzen suggests that TPB is open to further expansion and that the addition of potential predictors can enhance the model's explanatory power (26). Several studies on business and sports suggest that perceived availability might be associated with behavior, although they yield inconsistent results (30–34). Perceived availability represents the degree of accessibility to a particular entity, in our study context, cannabis, perceived by a person. We postulate that the factor can add to the explanatory power of our behavioral model in the context of cannabis use.

In Hong Kong, around 40% of the initiation of cannabis use happened at the age of 16–20 (35). Most students are admitted to college or university at the ages of 17 and 18. Studies show that living on campuses, attending university in urban geographic locations, and school climate are risk factors for cannabis initiation and use (36). Hence, students receiving tertiary education may have higher risks of cannabis use, warranting more public health investigation and interventions.

Epidemiological data in Hong Kong centre on the use of illicit drugs in general. To our knowledge, no local studies focus especially on cannabis and the factors associated with its use. We targeted undergraduates from the Faculty of Medicine of the Chinese University of Hong Kong (CUHK), the largest tertiary education institution in Hong Kong. The target population represents an age group prone to cannabis use. Understanding how future healthcare professionals conceptualise cannabis would be important because they may have discursive power on the image of cannabis, influencing public perceptions. However, literature in this regard is inadequate (37). Along this line of thought, we assess their knowledge of cannabis, in addition to TPB and potential constructs.

Overall, we aim to

(1) Explore the proportion and determinants of cannabis use intention among university students with a health-related major.
(2) Assess the explanatory power of TPB constructs regarding the intention to use cannabis in the sample as specified.
(3) Assess knowledge and perceived availability about cannabis use as additional factors for TPB and cannabis use intention.

## Materials and methods

### Participants and data collection

Our data were obtained from a cross-sectional survey. Due to the prolonged suspension of face-to-face classes amid COVID-19, we visited online classes for survey distribution and collection.

Prior to official data collection, a visit plan was devised to cover all students from the faculty. We chose one major-required course for each year of study and each major. Some batches of students did not have major-required courses. Multiple elective courses were visited. Exceptions were Bachelor of Nursing and Bachelor of Science in Biomedical Sciences, for which most of the class visits requests could not be entertained. For these two majors, mass mail was sent. Access to the survey was in the form of a link and QR code. Survey objectives and information for consent are shown adjacent to the link and verbally explained. The data collection period lasted from 28^th^ August to 12^th^ October. Completion of the survey took ~10 min. Survey responses were captured in Qualtrics, which prevented multiple participation by using cookies and converted responses into a dataset. All undergraduate students from the Faculty of Medicine of CUHK were eligible.

A sample size of 546 is estimated with the proportion of intention to be ~16.5% (38), where nine variables are to be included in the multiple logistic regression. A total of 996 respondents participated. Six hundred twenty-nine responses were complete and included for analysis. The response rate was 629/996 × 100% = 63.2%. The demographic and social backgrounds of respondents are summarised in Table 1. About 15% of respondents showed intention to use cannabis. 59.0% of respondents were female. The most frequently reported age group was 18 (22.6%), followed tightly by 21 (19.7%), 19 (18.4%), and 20 (17.5%). The age group was also the only demographic factor associated with intention (*p* < 0.05). Respondents pursuing a Bachelor of Medicine and Bachelor of Surgery made up the greatest proportion of all (44.4%), while Bachelor of Science in Biomedical Sciences made up the least (3.3%).

**Table 1:** Characteristics of respondents.

		**(*****n*** = **629)**		
	**Total (*n*, %)**	**Crude OR**	**95% CI**	***p*-values**
**Intention**				
Yes	92 (14.6)	N/A	N/A	N/A
No	537 (85.4)	N/A	N/A	N/A
**Sex**				
Female	371 (59.0)	1	1	N/A
Male	258 (41.0)	1.38	0.89–2.16	0.152
Age^*^				0.029
17	36 (5.7)	1	1	N/A
18	142 (22.6)	0.61	0.18–2.06	0.422
19	116 (18.4)	0.92	0.28–3.06	0.896
20	110 (17.5)	2.12	0.68–6.59	0.196
21	124 (19.7)	1.92	0.62–5.95	0.258
22	51 (8.1)	1.95	0.56–6.80	0.294
23	23 (3.7)	1.20	0.24–5.93	0.823
24 or above	27 (4.3)	2.29	0.58–9.08	0.240
**Major**				
Bachelor of Chinese Medicine	50 (7.9)	1	1	0.966
Bachelor of Medicine and Bachelor of Surgery	279 (44.4)	0.93	0.41–2.12	0.864
Bachelor of Nursing	97 (15.4)	0.96	0.38–2.45	0.932
Bachelor of Pharmacy	81 (12.9)	0.66	0.24–1.83	0.421
Bachelor of Science in Biomedical Sciences	21 (3.3)	0.55	0.11–2.85	0.479
Bachelor of Science in Community Health Practice	27 (4.3)	0.66	0.16–2.71	0.561
Bachelor of Science in Gerontology	28 (4.5)	1.14	0.33–3.90	0.833
Bachelor of Science in Public Health	43 (6.8)	1.20	0.41–3.53	0.740
Prefer not to say	3 (0.5)	N/A	N/A	N/A
**Stayed outside HK for** **≥6 months in the past 10 years**				
**Yes**	86 (13.7)	1	1	
Recreational cannabis illegal	31 (36.0)	1		
Recreational cannabis legal	55 (64.0)	1.97	0.46–8.51	0.364
Medical cannabis illegal	75 (87.2)	1		
Medical cannabis legal	11 (12.8)	1.16	0.36–3.75	0.810
**No**	543 (86.3)	0.78	0.43–1.44	0.427
Lubben social network scale (Mean, SD)	16.91 (4.90)	1.00	0.96–1.05	0.969

* *p* < 0.05 by Chi-square test.

### Instrument

To ensure the feasibility of the data collection method and face validity of the questionnaire, a pilot involving 48 subjects recruited by convenience sampling in the same population was done in April 2020. Face validity was established by assuring the clarity and meaning of the questions. Reliabilities of constructs composed of more than one item were estimated with Cronbach's alpha in the final sample, where the attitude construct has 0.95 and knowledge has 0.35. The domain knowledge of cannabis is a formative construct, where a change in one subscale does not imply the same directional change in other subscales. Therefore, a low Cronbach's alpha indicates that the questions within the knowledge construct are not redundant and not homogenous (39).

#### Extended theory of planned behavior

For knowledge, questions were designed to assess understanding of the properties, health effects, and legal statuses of cannabis. For properties, participants were asked to state whether “analgesics,” “hallucinogens,” “depressants,” “stimulants,” “tranquiliser,” and “others,” describe the type(s) of substance to which cannabis belong. Then, participants were to tell whether the following were active ingredients, namely “THB,” “CBD,” “CHB,” “THC,” and “TCD.” A maximum of 6 and 5 marks can be scored from the two parts respectively. For health effects, we asked if cannabis could lead to “anxiety,” “depression,” “increased risk of Parkinson's disease,” “euphoria,” “delusions,” “increased salivation,” “sleeplessness,” “dizziness,” “decreased heart rate,” “schizophrenia,” and “abnormally high body temperature.” A maximum of 11 marks can be earned. For legal statuses, we asked whether medical and recreational cannabis were respectively legal at the time of survey entry, in a range of states including Canada, Mainland China, United Kingdom, Japan, South Korea, Macau, Malaysia, South Africa, Hong Kong, Taiwan, and Singapore. A maximum of 11 marks can be earned, where each correct item accounts for 0.5 marks. The full marks for the total knowledge score are 33. Wrong responses, non-responses, and uncertainty would not result in mark deductions.

For attitude, we prepared sixteen questions listed in Table 2. Sixteen statements were rated by respondents. The questions were adapted from the Beliefs and Attitudes of Substance Abuse Inventory (BASAI), an instrument developed by Fok et al. to measure Chinese adolescent beliefs and attitudes towards substance abuse (40). Permission was granted by the authors. The items asked for the perceived effects of cannabis on mental, social, and general health. They also assessed the potential motivators of cannabis use, such as whether it helps “make friends,” “reduce anxiety,” “improve the quality of sleep,” and whether it is “fashionable” and “fun.” For each individual, scores from the sixteen items were added up to form the total attitude score.

**Table 2:** Cannabis knowledge and attitude of respondents stratified by intention.

	**(*****n*** = **629)**		**(*****n*** = **92)**		**(*****n*** = **537)**		
	**Mean**	**SD**	**Mean**	**SD**	**Mean**	**SD**	
Total knowledge score	16.65	4.29	17.84	4.64	16.56	4.20	0.004
Properties score	6.58	1.42	6.90	1.64	6.53	1.38	0.019
Health effects score	4.47	2.07	4.95	2.12	4.39	2.05	0.021
Legal statuses score	5.60	2.80	5.99	2.73	5.53	2.81	0.147
Total attitude score^a^	34.89	14.28	57.97	10.52	30.93	10.63	<0.001
Using cannabis will not affect one's behavior	1.82	1.12	3.38	1.45	1.55	0.80	<0.001
Using cannabis will adversely affect my daily activities^a^	3.54	1.23	2.51	1.18	3.72	1.15	<0.001
Using cannabis helps me make friends	1.80	1.18	3.58	1.36	1.50	0.83	<0.001
Using cannabis is fashionable	1.80	1.19	3.63	1.36	1.49	0.83	<0.001
I think that cannabis will reduce anxiety	2.64	1.18	3.51	1.01	2.49	1.14	<0.001
Using cannabis will improve the quality of my sleep	2.20	1.12	3.53	0.95	1.97	0.98	<0.001
Using cannabis is fun	2.14	1.28	4.09	0.91	1.80	1.01	<0.001
I believe that cannabis has many positive effects	2.50	1.18	3.72	0.95	2.30	1.09	<0.001
I believe that cannabis has many negative effects^a^	3.66	1.20	2.36	1.10	3.88	1.07	<0.001
I believe that cannabis will not be harmful to one's health	2.08	1.21	3.77	1.14	1.79	0.96	<0.001
I believe that cannabis will not lead to dependence	2.10	1.24	3.59	1.22	1.84	1.05	<0.001
Using cannabis will not worsen my memory	1.96	1.10	3.58	1.17	1.68	0.81	<0.001
I believe that cannabis will only affect my mental state for a short period of time	2.30	1.16	3.64	1.02	2.07	1.02	<0.001
Using cannabis will affect my judgement^a^	3.82	1.12	2.82	1.22	3.99	1.01	<0.001
Using cannabis occasionally will not be harmful to me	2.10	1.25	3.88	1.15	1.80	0.99	<0.001
Cannabis should not be harmful if I just use it once	2.46	1.30	3.76	1.10	2.24	1.20	<0.001

a In the calculation of means total attitude score, three attitude items' coding are reversed as the views potentially reduce intention to use cannabis. In particular, “strongly disagree” becomes 5 and “strongly agree” becomes 1.

To assess the perceived behavioral control (PBC), the subjects were asked to rate the statement, “if I use cannabis, I will have the confidence to stop using them at any time.” Two kinds of norms were measured, including the descriptive norm among young people (“I think that cannabis use is common among young people”) and the injunctive norm from the society (“I think Hong Kong society is against the use of cannabis”).

For perceived availability, respondents were asked to rate “I think cannabis is readily available in my community.”

The core outcome of this study, the intention to use cannabis, was measured by asking respondents to rate the extent to which they agree with “I plan to use cannabis in the future.”

All items above were assessed with a Likert scale from 1 to 5, with 1 being “strongly disagree,” 3 being “neutral,” and 5 being “strongly agree.” Most attitude statements favoured cannabis, while some items disfavoured. The latter were reversely coded for the calculation of the total attitude score, where “strongly disagree” became “5” and “strongly agree” became “1.”

#### Demographics

Basic information, including subjects' sex, age, and major, was obtained. We collected geographical data, including whether the subjects had stayed outside Hong Kong for at least 6 months, the legal statuses of cannabis in the places they stayed, and that of cannabis for the nationality they identified with. In addition, we measured their social network with Lubben Social Network Scale-6 (final sample Cronbach's alpha: 0.798) (41), a validated tool that indicates social ties, with kind permission from the authors. The total scale, ranging from 0 to 30, is compiled by 6 items with equal weighting. A higher score indicates a better social network. These are potential confounders to be controlled.

### Statistical analysis

Demographic variables were respectively tested for their association with intention with logistic regression, except for Lubben Social Network Scale-6 compiled with a *T*-test. Variables with statistical significance indicated an association with intention and are potential confounders. They were controlled in our model.

Participants rating 4–5 for intention statement were categorised as the “with intention” group, whereas those rating 1–3 as the “without intention” group.

Finally, the associations between interested independent variables and the intention were assessed. Crude odds ratios for each variable were computed by logistic regressions. To compile adjusted odds ratios, statistically significant demographic variables were assessed as covariates for each independent variable. A TPB-driven instead of a data-driven approach was used when building the two logistic regression models, i.e., the core model and the extended model. Several critical assumptions underlying multiple logistic regression were tested. Box-Tidwell Test was utilised to confirm linearity in the logit for continuous variables. All continuous independent variables (knowledge, PBC, norms, and attitudes) have a linear relationship with the logit of intention. Hence, the assumption was not violated. When using a variance inflation factor (VIF) threshold of 2.5, there was no multicollinearity between independent variables in the models. No outliers have a z-score larger than 3. All other assumptions were met.

To understand whether attitude moderates the relationship between PBC and intention, we did an exploratory *post-hoc* analysis by stratifying the samples into two groups, one with a positive attitude and the other with a negative or neutral attitude. A positive attitude was defined as having a total attitude score of 1 SD above the mean. We then see if PBC and intention were associated in each of the two groups.

A *p*-value smaller than 0.05 was taken as the level for statistical significance.

IBM SPSS Statistics Desktop 26.0 (IBM Corp., Armonk, N.Y., the U.S.) and Statgraphics Centurion 19 (Statgraphics Technologies, Inc., The Plains, Virginia, the U.S.) were used for data analysis.

### Ethical statement

Ethics approval was obtained from The Joint Chinese University of Hong Kong – New Territories East Cluster Clinical Research Ethics Committee (CRE2019.496). Research reporting was on overall data at an aggregate level. No personally identifiable information was collected. The data was saved in a password-protected online account and computer.

## Results

The knowledge and attitude of respondents regarding cannabis and its use are presented in Table 2. The mean Total Knowledge Score was 16.65 (SD = 4.29). Respondents showing intention to use cannabis had a slightly higher mean Total Knowledge Score (17.84, SD = 4.64) than those without intention (16.56, SD = 4.20, p < 0.01). Respondents with intention consistently showed higher means in all knowledge components, including Properties Score (6.90, SD = 1.64 vs. 6.53, SD = 1.38, *p* < 0.05), Health Effects Score (4.95, SD = 2.12 vs. 4.39, SD = 2.05, *p* < 0.05), and Legal Statuses Score (5.99, SD = 2.73 vs. 5.53, SD = 2.81, *p* = 0.147), although the last of which lacked statistical significance.

The mean Total Attitude Score was 34.89 (SD = 2.80). Respondents with an intention to use cannabis (57.97, SD = 10.52) had almost double the mean Total Attitude Score of those without intention (30.93, SD = 10.63, *p* < 0.001). The three attitude items disfavouring cannabis all showed lower means among respondents with an intention to use cannabis, whereas the rest of the items consistently showed higher means (*p* < 0.001).

Univariate analyses of each key construct are listed in Table 3. Of note, perceived behavioral control gave the highest magnitude of association (COR = 3.44, 95% CI 2.72–4.35, *p* < 0.001). A higher Total Attitude Score increased the odds of intention (COR = 1.23, 95% CI 1.18–1.28, *p* < 0.001). Both injunctive norm (COR = 0.51, 95% CI 0.42–0.61, *p* < 0.001) and descriptive norm (COR = 2.24, 95% CI 1.81–2.77, *p* < 0.001) were significant. Perceived Availability also carried a significant association with intention (COR = 2.75, 95% CI 2.22–3.40, *p* < 0.001). An increase in one mark in Total Knowledge Score increased the odds of intention by 1.08 (COR = 1.08, 95% CI 1.03–1.14, *p* < 0.01). All TPB and additional constructs were individually associated with the intention to use cannabis.

**Table 3:** Univariate analysis of key constructs with cannabis use intention.

**COR (95% CI)**	***p*-value**	**AOR (95% CI)**	***p*-value**
Perceived behavioral control	3.44 (2.72–4.35)	<0.001	3.38 (2.66–4.30)	<0.001
Descriptive norm	2.24 (1.81–2.77)	<0.001	2.20 (1.76–2.75)	<0.001
Injunctive norm	0.51 (0.42–0.61)	<0.001	0.50 (0.41–0.61)	<0.001
Total attitude score	1.23 (1.18–1.28)	<0.001	1.24 (1.19–1.30)	<0.001
Total knowledge score	1.08 (1.03–1.14)	0.004	1.07 (1.02–1.13)	0.013
Perceived availability	2.75 (2.22–3.40)	<0.001	2.79 (2.24–3.49)	<0.001

COR, crude odds ratio.

AOR, age-adjusted odds ratio.

Table 4 puts together the core TPB model (Nagelkerke R^2^ = 0.706) and its extended version (Nagelkerke R^2^ = 0.736) for cannabis use intention, where both models indicate good fits. In the core model, only descriptive norm (AOR = 1.59, 95% CI 1.12–2.27, *p* < 0.05) and attitude (AOR = 1.19, 95% CI 1.14-1.25. p < 0.001) demonstrate statistically significant correlation with intention. With the addition of knowledge and perceived availability, the descriptive norm loses its statistical significance, but still carries a higher explanatory power than the injunctive norm (standardized coefficients: 0.324 vs. −0.143). Attitude (AOR = 1.19, 95% CI 1.13–1.25, *p* < 0.001) becomes the only core TPB construct showing association with intention. Among the supplemented variables, perceived availability (p < 0.005) has a considerable association with intention. Treating the highest rating to perceived availability as the reference group, those who disagreed that cannabis was readily available had a much lower chance of having intention (AOR = 0.16, 95% CI 0.03–0.80, *p* < 0.05). Moreover, perceived availability carries a particularly high explanatory power in the model (standardized coefficient = 0.570), only second to attitude (standardized coefficient = 2.433). Finally, knowledge (AOR = 1.07, 95% CI 0.98–1.18, *p* = 0.152) is not shown to be associated with intention.

**Table 4:** Expanded theory of planned behavior model for cannabis use intention.

**B**	**S.E.**	**Standardized coefficient**	**df**	**Sig.**	**AOR (95% CI)**	**Nagelkerke R^2^**
**Core model**							0.706
PBC	0.340	0.199	0.462	1	0.088	1.40 (0.95–2.07)	
Descriptive norm	0.464	0.181	0.511	1	0.010	1.59 (1.12–2.27)	
Injunctive norm	−0.291	0.162	−0.333	1	0.073	0.75 (0.54–1.03)	
Attitude	0.177	0.025	2.426	1	0.000	1.19 (1.14–1.25)	
**Extended model**							0.736
PBC	0.363	0.205	0.470	1	0.076	1.44 (0.96–2.15)	
Descriptive norm	0.345	0.192	0.324	1	0.073	1.41 (0.97–2.06)	
Injunctive norm	−0.191	0.189	−0.143	1	0.311	0.83 (0.57–1.20)	
Attitude	0.173	0.026	2.433	1	0.000	1.19 (1.13–1.25)	
Knowledge	0.069	0.048	0.302	1	0.152	1.07 (0.98–1.18)	
**Perceived availability**			0.570	4	0.004		
Very disagree	−1.529	0.891	/	1	0.086	0.22 (0.04–1.24)	
Disagree	−1.854	0.832	/	1	0.026	0.16 (0.03–0.80)	
Neutral	−1.845	0.794	/	1	0.020	0.16 (0.03–0.75)	
Agree	−0.141	0.811	/	1	0.862	0.87 (0.18–4.26)	

PBC, Perceived behavioral control.

Demographic confounder (age) is controlled.

Further examination of PBC stratified by respondents' total attitude score is summarised in Table 5. PBC showed a significant association with intention among respondents with negative to neutral attitudes towards cannabis (COR = 2.67, 95% CI 1.76–4.04, *p* < 0.001), but not so among those with a positive attitude.

**Table 5:** Association between perceived behavioral control an intention stratified by total attitude score.

**Crude OR**	**95% CI**	***p*-value**	**AOR**	**95% CI**	***p*-value**
Negative-neutral	2.67	1.76, 4.04	<0.001	2.48	1.63, 3.77	<0.001
Positive	1.32	0.85, 2.05	0.213	1.35	0.85, 2.14	0.205

## Discussion

Against the backdrop of cannabis legalization and popularization, we have conducted a cross-sectional study in Hong Kong, aiming to explore the determinants of cannabis use intention, with a particular focus on the theory of planned behavior. Our sample consists of a population rarely seen in cannabis studies, which was predominantly students of different allied health professionals. In our sample, each TPB variable alone significantly correlated to the intention to use cannabis. When placed in the extended regression model, all variables except attitude and perceived availability were no longer significant in the presence of other covariates. Comparing the two norms, the descriptive norm was consistently more influential than the injunctive norm, although both have not demonstrated statistical significance in our final model. Intention and knowledge were individually correlated but were not so in the final model. Overall, our study indicates patterns of cannabis use intention among young future healthcare workers, who are potential influencers of public health practice. It added to the small body of cannabis literature in cannabis-illegal regions and was the first study in Hong Kong investigating cannabis use intention and its determinants.

Previous research found that freshmen were the most prominent high-risk group for drug abuse, probably due to the “college effect” under which they have higher stress and responsibility, life transition, and a higher degree of freedom in a new community (42, 43). Contrary to this finding, we identified a peak of cannabis use intention among those aged 20–21, as freshmen in Hong Kong universities are typically aged 17–18. The development of social networks in college could increase the risk of cannabis use intention as this may expose students to resources of cannabis. Therefore, the authors speculate that a combination of the college effect and the aforementioned effect account for the peak in this age group. Moreover, some of those aged 20–21 could have been admitted from a previous higher diploma or associate degree, and the transition to a new institution may have put them at risk. Other underlying possibilities include larger stress due to seniority and cultural differences between Hong Kong and the West.

Knowledge of respondents was assessed according to the best available evidence we had at the time of data analysis. Nevertheless, cannabis research is rapidly evolving, and the knowledge encompassing it is subjected to change.

Subjects with an intention to use cannabis were more knowledgeable about cannabis. However, the score differences between subjects with and without intention were small, and the association between knowledge and intention was weak. The small score difference detected by our relatively large sample size could imply that it is only of statistical but not practical importance. However, it does challenge the a priori presumption, namely, that a higher intention to use a substance should be associated with a relative lack of knowledge. Moreover, the mean difference in the Legal Statuses score is not even statistically significant. These show the limited interaction between knowledge and cannabis use intention.

Few studies have investigated the relationship between knowledge of cannabis and intention to use. Among these limited studies, a Thai study has identified knowledge as a protective factor for the intention to take medical cannabis, running contrary to our results (44). We hypothesize that contextual differences may explain the discrepancy. At the time of their study, Thailand had just legalized medical cannabis. Before the commencement of this present study, Hong Kong has yet to legalize medical and recreational cannabis, and public health advocacy is generally unfavourable of cannabis use. Besides, the two studies have distinct study populations. In Rakpanich et al.'s study, respondents were rural Thais who were married and had low education levels (44). In our study, most participants were young and so were unlikely to be married, given the average ages of marriage in Hong Kong were 29.4 and 31.4 for females and males, respectively (45). Our participants also had higher education levels and lived in urban areas.

Interestingly, knowledge as one of the conventional triads (knowledge, attitude, and practice) for devising public health interventions is not necessarily reflective of intention, as shown in our study. This phenomenon could be unique to cannabis, where the same pieces of knowledge can be differentially interpreted. For example, there are both objective and subjective components in the evaluation of harm. Being more knowledgeable can both encourage or discourage cannabis use when there is controversy over how evidence is understood. Public health interventions shall consider the exact role of knowledge when it is targeted for behavioral modifications.

Similar to other studies (21, 23, 46, 47) and as expected, the total attitude score was positively associated with intention. Directions of association were consistent in all attitude items, and their aggregate attitude towards cannabis correlates with intention. Most importantly, attitude is the only core TPB construct retaining the significant association with intention upon the addition of potential covariates.

All the other TPB constructs correlated individually with intention. In both the core and extended models, the descriptive norm was consistently more influential than the injunctive norm based on the standardized coefficients. The descriptive norm has more explanatory power (higher Odds Ratio or explains more variance) than the injunctive norm, as shown in previous studies on the descriptive norm (23) and meta-analysis (48). In this meta-analysis (48), the descriptive norm has a particularly closer relationship with the behavior of adolescents and school-aged compared to adults (descriptive norm is better correlated with behavior in younger population). Such findings are different from that of Ito et al., who showed that only the injunctive norm, but not the descriptive norm, was a correlate. It could be explained by the differences in study populations. Ito et al. sampled students from the University of Colorado, where the baseline cannabis use rate (38.2%) was high, and the community was pro-marijuana. Our target population, however, has a low baseline cannabis use rate (6.4%). Hence, the effect of the descriptive norm may be less significant in their study.

Upon *post-hoc* analysis, PBC correlated well with intention univariately. However, when put alongside other variables in the model, it became insignificant. Therefore, we further investigated the moderation effect of attitude on PBC and intention, as suggested by Conner and McMillan. Previous studies (23, 49) showed a *negative* association between PBC and intention for those having a negative or neutral attitude towards cannabis. For those with a positive attitude, no association was observed. Our results resemble part of those studies. There was indeed no association between PBC and intention when focusing on those with a positive attitude. However, there was a *positive* association between those having a negative or neutral attitude. It should be unrelated to social desirability because, as Conner and McMillan suggested (23), if social desirability is involved, there should be a positive correlation between PBC and intention among those with a positive attitude, while no correlations between the variables would be observed for those having a neutral or negative attitude. Hence, similar to what Umeh and Patel suggested, we proposed that the discrepancy could be due to differences in intention measurement (50). Conner and McMillan measured the intention *not* to use cannabis while we measured the intention to use cannabis.

Perceived availability was found to carry a potent influence on intention to use cannabis, only second to attitude. It differs from other studies, where the exact influences of perceived availability are either not reported or contradictory (32–34). The relationship between perceived availability and drug use was explored in Hong Kong 20 years ago and was found to be closely related to cannabis use (51). The study sample (*N* = 969) consisted of 59.8% secondary school students and 40.2% incarcerated delinquents.

Ajzen (26) suggested that the availability of time is a control belief, a sub-construct of PBC. Analogous to his line of thoughts, we postulate that perceived availability is also a control belief closely related to PBC, hence explaining its strong effect on intention. Another possibility is that perceived availability could be related to the descriptive norm. If cannabis consumption is perceived to be prevalent among peers, the participant may also perceive cannabis as readily available in the participant's community. Our third postulation is that perceived availability was indicative of cannabis use. Those who use cannabis know where to obtain cannabis, so they likely have more access to it. In addition, the intention is very indicative of use [meta-analysis shows that 43–62% of intention will be translated into behavior (27)], so it is reasonable to assume a correlation between perceived availability and intention.

In this study, Nagelkerke R^2^ was used to describe the model fit. However, one should be aware that R^2^ gain might be due to the addition of new variables *per se*, not necessarily a better explanatory power.

### Limitations

Our study was designed before the outbreak of COVID-19 and therefore does not consider the potential effects of the pandemic. Some studies showed that confinement in this period might raise the intention to use cannabis (52, 53). The results may differ considerably from a normal scenario. We suggest that readers interpret the results with scrutiny.

A small, convenient, and non-random sample was used for this study, undermining its representativeness to future health care workers. Also, there could be some mismatch between sample makeup and the target population. Only a small portion of participants was nursing students in our sample, but the proportion should be much larger in the Faculty of Medicine. Moreover, it is not generalisable to other educational institutions in Hong Kong. It also limits the generalisability to the future healthcare workforce in Hong Kong, as more than half of the workforce are nurses (54).

We might have underreported the prevalence of intention and use, as those who use or intend to use cannabis may miss out on class (55, 56). Bias may arise from self-report, social desirability, and memory inaccuracies.

It has been argued that cannabis use starts to develop at a much younger age (15–16 years), then increases with age, and finally tapers off (57, 58). Also noteworthy is that the younger is particularly prone to risky behaviors. In light of this, our sample may not be able to capture the initial phase of cannabis use and the characteristics of younger populations.

As an inherent limitation of a cross-sectional survey, we cannot establish any temporal relations, not to mention causal relations between variables. Further studies may work on these limitations, along with the moral norm, societal descriptive norm, and peer injunctive norm, which we have not but are worth exploring in the Asian population.

### Implications

Overall, our findings both question and support the applicability of the TPB model for cannabis use and potentially other substance use contexts. On the positive side, the attitude has an immutable strength of association with intention. It shall continue to inform public health practices of the factors leading to cannabis use and targets of interventions. It would be beneficial to break down the processes whereby sociocultural contexts shape a population's perceptions and preferences of cannabis. For example, policymakers may identify the major sources of cannabis information, their contents, modes of delivery, and influences on different population groups. This is essential when cannabis and its culture become ever more globalized and that channels of information are unprecedentedly diverse. Unlike many other illicit drugs, cannabis is often commercialized as a recreational product with certain pharmaceutical effects, such as helping with sleep and anxiety. Even in Hong Kong, where THC is generally banned, there have been CBD products claiming the aforementioned effects. Therefore, how the population comes to interpret the functional aspects of cannabis and the underlying factors should be noteworthy for policymakers. This certainly does not mean that the psychosocial aspects of cannabis can be neglected. It is essential to keep inquiring into the role of cannabis in self-identity and relationships. While our study has not indicated an association between social networks and cannabis use intention, cannabis can still be perceived to play some role in social networks, especially among peers. In addition, our results encourage experimentation on attitude-modifying interventions, to which more resources can be directed. Equally important is to review current health promotion and education programs with regard to their relative position and effects among all other determinants of attitude. Policymakers should keep programs up to date in terms of their substances and modalities, especially in response to cultural influences and shifts among younger generations.

On the sceptical side, however, our findings imply uncertainties over the value of other TPB constructs. Many of the constructs have not shown significant associations with intention, and the types of norms included produce different results. Adding to the complexity is the potential of perceived availability and its effects on the TPB constructs. The implications are twofold. First, researchers shall continue to explore the application of various behavioral health models in a specific context and population. In addition to TPB, other health psychology models, such as the health belief model and social cognitive theory are also potential candidates when it comes to understanding health behaviors. Second, public health campaigns targeting cannabis norms have to distinguish the types of norms involved in light of their heterogeneous explanatory powers for intention. Third, a more in-depth delineation of perceived availability and its relations to TPB constructs is warranted.

## Conclusion

This study demonstrates that TPB has limited explanatory power for cannabis use intention among a group of Hong Kong university students. Further research efforts may explore whether an extension of the model is appropriate for the context of cannabis. Comparisons can be made between various behavioral health models for the same population as well.

## Author's note

The data collection and analysis was done during PC is employment under The Chinese University of Hong Kong.

## Data availability statement

The original contributions presented in the study are included in the article/supplementary files, further inquiries can be directed to the corresponding author.

## Ethics statement

The studies involving human participants were reviewed and approved by the Joint Chinese University of Hong Kong – New Territories East Cluster Clinical Research Ethics Committee. Written informed consent from the participants' legal guardian/next of kin was not required to participate in this study in accordance with the national legislation and the institutional requirements.

## Author contributions

SH and YW: conceptualisation, data curation, formal analysis, methodology, project administration, writing—original draft, and writing—review and editing. PC: participant recruitment and writing—review and editing. All authors contributed to the article and approved the submitted version.

## Conflict of interest

The authors declare that the research was conducted in the absence of any commercial or financial relationships that could be construed as a potential conflict of interest.

## Publisher's note

All claims expressed in this article are solely those of the authors and do not necessarily represent those of their affiliated organizations, or those of the publisher, the editors and the reviewers. Any product that may be evaluated in this article, or claim that may be made by its manufacturer, is not guaranteed or endorsed by the publisher.
